# Diagnostic value of urinary mevalonic acid excretion in patietns with a clinical suspicion of mevalonate kinase deficiency (MKD)

**DOI:** 10.1186/1546-0096-13-S1-O57

**Published:** 2015-09-28

**Authors:** J Jeyaratnam, N ter Haar, M de Sain-van der Velden, H Waterham, M van Gijn, J Frenkel

**Affiliations:** 1 University Medical Center Utrecht, Department of Pediatrics, Utrecht, Netherlands; 2 University Medical Center Utrecht, Laboratory for Translational Immunology, Utrecht, Netherlands; 3 University Medical Center Utrecht, Department of Medical Genetics, Utrecht, Netherlands; 4 Academic Medical Center, Clinical Chemistry and Pediatrics, Amsterdam, Netherlands

## Introduction

Mevalonate kinase deficiency (MKD) is a rare hereditary autoinflammatory syndrome, characterized by recurrent fever episodes with gastrointestinal complaints, rash and arthralgia. In patients suffering from MKD, the reduced enzyme activity leads to an accumulation of mevalonic acid which is excreted in the urine. Therefore, an elevated mevalonic acid excretion is suggestive of MKD. However, the diagnostic value of this analysis has not been investigated yet and remains unclear.

## Objectives

To investigate the diagnostic value of urinary mevalonic acid excretion in patients with suspected MKD.

## Patients and methods

In this retrospective analysis, all patients in whom both measurement of mevalonic acid and genetic testing had been performed in the preceding 17 years have been included. Samples were analyzed by using gas chromatography-mass spectrometry (GC-MS) and concentrations were expressed as mmol/mol creatinine. The excretion of mevalonic acid was compared with age dependent reference values, validated at our hospital. The presence of two pathogenic *MVK* mutations was considered to be the gold standard for the diagnosis of MKD.

## Results

This study included 62 patients (33 male, 29 female, aged: 0-36 year) with clinical features suggestive of MKD.

Thirteen patients harboured two *MVK* mutations, twelve of them excreted elevated amounts of mevalonic acid. In one patient mevalonic acid could not be detected, despite the fact that urine was collected during a febrile episode. Six patients had an elevated mevalonic acid excretion, but harboured no *MVK* mutations. However, repeated measurements in all six patients were ultimately normal.

This resulted in a sensitivity of 92%, a specificity of 88%, a positive predictive value of 68% and a negative predictive value of 98%. The positive likelihood ratio is 7.7 and the negative likelihood ratio is 0.09.

## Conclusion

MKD seems very unlikely in patients with a normal mevalonic acid excretion, but it cannot be excluded completely. Furthermore, a positive urinary mevalonic acid excretion requires *MVK* analysis to confirm the diagnosis MKD. However, as long as genetic testing is not widely available and affordable, measurement of urinary mevalonic acid is a fair way to select patients for *MVK*-gene analysis.

